# Supplementation of Flaxseed Increases Omega-3 Fatty Acids Accumulation in the Meat of Beijing-You Chickens

**DOI:** 10.3390/vetsci13020128

**Published:** 2026-01-28

**Authors:** Wei Wang, Ming Jia, Xianren Jiang, Danyang Zhao, Jie Wang, Ting Yao, Bo Zhang, Junguo Li, Zhiying Fan, Xu Gu

**Affiliations:** 1 Institute of Feed Research of Chinese Academy of Agricultural Sciences, Beijing 100081, China; w15633753273@163.com (W.W.); jiaming@caas.cn (M.J.); jiangxianren@caas.cn (X.J.); zhaodanyang0912@163.com (D.Z.); wangjie@caas.cn (J.W.); zhangbo@caas.cn (B.Z.); lijunguo@caas.cn (J.L.); fanzhiying@caas.cn (Z.F.); 2 Laboratory of Quality & Safety Risk Assessment for Animal Products on Feed and Feed Additive Factors, Ministry of Agriculture and Rural Affairs, Beijing 100081, China; 3 Beijing Municipal Veterinary Drug and Feed Monitoring Center, Beijing 101100, China; yaoting@126.com

**Keywords:** flaxseed, Beijing-you chickens, omega-3 polyunsaturated fatty acids

## Abstract

This study investigated the effects of different dietary flaxseed inclusion levels (0, 2, 4, 6, 8, and 10%) on the fatty acid composition, plasma biochemical parameters, and meat quality of Beijing-you chickens. A total of 960 6-week-old birds were randomly assigned to six dietary treatments in a completely randomized design, with eight replicates per treatment. Dietary flaxseed supplementation significantly decreased plasma alanine aminotransferase (ALT), aspartate aminotransferase (AST), total cholesterol (TC), and triglyceride (TG) concentrations (*p* < 0.05), while significantly increasing high-density lipoprotein cholesterol (HDL-C) levels (*p* < 0.05). Meat quality was also improved, as evidenced by reduced drip loss *(p* < 0.05). Moreover, gas chromatography analysis revealed that increasing dietary flaxseed levels markedly elevated the concentrations of total ω-3 polyunsaturated fatty acids (ω-3 PUFAs) in both breast and leg muscles (*p* < 0.05), accompanied by a significant reduction in the ω-6/ω-3 ratio (*p* < 0.05). Dietary flaxseed supplementation did not significantly affect the docosahexaenoic acid (DHA) content in pectoral muscle (*p* > 0.05), with the highest value observed at the 6% inclusion level. In conclusion, dietary flaxseed supplementation effectively enriches ω-3 PUFAs in chicken meat while simultaneously enhancing metabolic health indicators and improving meat quality traits.

## Simple Summary

Beijing-you chickens, a valuable local Chinese breed, are prized for their tender meat and naturally higher polyunsaturated fatty acids (PUFAs) compared to commercial breeds. Flaxseed is also a rich source of PUFAs, making it a promising dietary supplement to enhance poultry meat’s nutritional value. This study aimed to explore how different levels of dietary flaxseed affect PUFA deposition in Beijing-you chickens. Results showed that 8% flaxseed supplementation for 12 weeks achieved the highest PUFA deposition in muscle while improving the birds’ health and meat quality. In conclusion, this research provides theoretical and technical support for practical poultry production, helping farmers meet consumer demand for healthier, nutrient-dense meat and promoting the development of local breed resources.

## 1. Introduction

Flaxseed is rich in fat (29–41%), protein (21–31%), and crude fibre (5–10%) on a dry matter (DM) basis [1]. When incorporated at appropriate levels into livestock and poultry diets, it does not compromise production performance and can enhance the omega-3 polyunsaturated fatty acids (ω-3 PUFAs) content of animal products while supporting immune function [2,3]. Both monounsaturated and polyunsaturated fatty acids, particularly ω-3 PUFAs, are widely recognized for their nutritional benefits [4]. Health authorities generally recommend a daily ω-3 PUFAs intake of 1.4–2.5 g, including 140–600 mg of combined eicosapentaenoic acid (C20:5n-3, EPA) and docosahexaenoic acid (C22:6n-3, DHA) [5]. In animals, ω-3 PUFAs deposition occurs primarily through two pathways: dietary α-linolenic acid (ALA) may be progressively converted to EPA and DHA, or preformed long-chain ω-3 PUFAs may be directly absorbed and stored [6]. Previous studies have demonstrated that supplementing poultry diets with flaxseed or flaxseed oil increases linoleic acid (ω-6) and linolenic acid (ω-3) concentrations in muscle, thereby elevating overall PUFA levels [7]. However, flaxseed also contains antinutritional factors such as cyanogenic glycosides, which can release toxic hydrocyanic acid upon hydrolysis and pose potential health risks [8]. Therefore, the inclusion level must be carefully controlled in practical feeding applications.

Beijing-you chicken is a traditional breed originating from Beijing and recognized as a geographical indication product. This breed is prized for its meat quality, marked by higher intramuscular fat (typically 3–5%) and a distinct flavor profile enriched with compounds such as inosinic and glutamic acids [9]. Compared with commercial broilers, Beijing-you chickens exhibit higher PUFA levels—particularly ω-3 PUFAs—as well as greater concentrations of water-soluble flavour precursors, contributing to a richer broth aroma [10]. In addition, indigenous breeds such as Beijing-you generally exhibit a more desirable pattern of intramuscular fat deposition (i.e., moderate content and more uniform distribution), together with reduced drip loss and improved muscle quality traits, which collectively contribute to better eating quality [11,12,13]. By contrast, although fast-growing commercial broilers provide an efficient protein source, their rapid growth frequently compromises meat quality by reducing protein hydrolysis potential and water-holding capacity. Consequently, these distinctive lipid deposition patterns and fatty acid profiles establish the Beijing-you chicken as a highly responsive model organism, particularly suited for elucidating the mechanisms governing ω-3 PUFA deposition and metabolism.

This study aimed to elucidate the effects of dietary flaxseed level and feeding duration on ω-3 PUFA deposition in Beijing-you chickens, a slow-growing indigenous breed with lipid metabolism characteristics conducive to ω-3 enrichment. We hypothesized that: (1) prolonged feeding would be particularly effective in enhancing ω-3 PUFA accumulation when combined with moderate, growth-compatible flaxseed inclusion levels; and (2) an optimal dose–time combination exists that maximizes ω-3 PUFA enrichment in meat while maintaining growth performance comparable to that of the unsupplemented control.

## 2. Materials and Methods

### 2.1. Animal Ethics Approval

The trial was conducted at the Nankou Experimental Center of the Chinese Academy of Agricultural Sciences, that is located in Beijing, China. The animal procedures in this study were approved by the Animal Ethics Committee of the Institute of Feed Research of the Chinese Academy of Agricultural Sciences (IFR-CAAS20180521).

### 2.2. Flaxseed Preparation

Ground flaxseed, selected for its high ω-3 PUFA content, was incorporated into the experimental diets as a supplemental ingredient. It was thoroughly blended with the basal feed components and processed into uniform pellets for administration. The detailed nutritional composition of the flaxseed is presented in Table 1.

### 2.3. Experimental Design and Management

A total of 960 healthy 6-week-old Beijing-you chickens with an average initial body weight of 598 ± 20 g were selected for the experiment. Prior to randomization, birds were stratified by sex; males and females were independently assigned random numbers using a computer-generated list and then evenly allocated to six experimental treatments. Each treatment included eight replicates (pens), with 20 chickens per replicate (10 males and 10 females; 1:1 sex ratio). Replicates were blocked according to cage position to minimize environmental confounding effects. The six experimental diets consisted of a basal control (CT) and diets supplemented with 2, 4, 6, 8, or 10% flaxseed (T1–T5), formulated to meet the nutritional requirements for slow-growing chickens, based on the NRC recommendations [14], and adjusted to suit the growth characteristics of Beijing-you chickens (proximate composition shown in Table 2). Crude protein [15], ether extract [16], moisture [17], and ash [18] were determined according to standard procedures, and fatty acid profiles were analysed using gas chromatography after lipid extraction and methylation [19]. All diets were pelleted, and feed and water were provided ad libitum throughout the 12-week experimental period. Birds were reared in cages (1.5 × 0.8 × 0.5 m; 0.06 m^2^/bird) without litter, under a lighting regimen of 16 h light and 8 h dark. Ambient temperature was gradually adjusted from 24–26 °C in week 1 to 20–22 °C in week 12, with relative humidity maintained at 55–65% and mechanical ventilation provided. At weeks 2, 4, 6, 8, 10, and 12 of the trial, eight chickens per treatment (with a 1:1 sex ratio) were randomly selected and slaughtered. Pectoral and leg muscles were collected immediately to determine their fatty acid composition. At the end of the 12-week feeding trial, two independent sets of chickens were slaughtered to address the two core objectives of this study. One set was dedicated to assessing the dose-dependent effects of dietary flaxseed inclusion on fatty acid composition. The other set provided the final time-point data for evaluating the time-dependent effects of feeding duration. All experimental procedures complied with the Broiler Husbandry Management Manual and the Regulations for the Management of Laboratory Animals.

### 2.4. Measurement of Growth Performance

On the final day of the experimental period, all chickens were individually weighed to determine final body weight. Individual body weights were also recorded at the beginning of the trial, allowing the calculation of average daily gain (ADG) on an individual basis. During the feeding trial, chickens were slaughtered at 2-week intervals to assess time-dependent effects. At each sampling point, eight chickens per treatment were selected across all pens. All birds were individually marked and sexed at the start of the experiment, and a balanced proportion of male and female chickens (approximately 1:1) was maintained for each slaughter batch. Due to the periodic removal of chickens for sampling, the male-to-female ratio and stocking density within each pen changed over time. Given the well-established sex-related differences in growth rate and feed intake, pen-based calculations of growth performance parameters that rely on feed intake measurements, including average daily feed intake (ADFI) and feed conversion ratio (FCR), were considered biologically confounded and therefore were not performed.

### 2.5. Measurement of Plasma Biochemical Parameters

At the end of the experimental period, one bird was randomly selected from each replicate pen for biochemical analysis (*n* = 8 per treatment). Blood was drawn according to standard procedures for venous blood collection. Plasma was harvested by centrifugation at 4000 rpm for 10 min at 4 °C and stored frozen until analysis. Serum alanine aminotransferase (ALT), aspartate aminotransferase (AST), total cholesterol (TC), triglyceride (TG), high-density lipoprotein cholesterol (HDL-C), low-density lipoprotein cholesterol (LDL-C) and non-esterified fatty acids (NEFA) levels were measured using the microplate reader (Bio-Tek, Burlington, VT, USA) in accordance with the manufacturer’s instructions. The kits were purchased from Nanjing Jiancheng Bioengineering Institute (Jiancheng, Nanjing, China).

### 2.6. Measurement of Meat Quality

Pectoralis major muscles were harvested from each of the eight chickens per treatment group immediately after slaughter, which was conducted subsequent to blood collection. Specifically, these muscle samples were used for the determination of meat quality parameters, including pH value, shear force, and drip loss. pH was measured at 45 min post-mortem. Three measurements were performed at different positions for each sample, and the mean pH value was calculated. Each meat sample was suspended in a sealed container in a refrigerator at 4 °C. The meat was weighed again to calculate drip loss, and defined as the loss in weight after 24 h. Drip loss was determined using the standard bag method, by measuring the sample weight before (W1) and after (W2) storage and was calculated as (W1 − W2)/W1 × 100%. The pectoral muscle samples were cut into strips (2 × 1 × 0.5 cm) along the muscle fiber direction, and shear force was determined using a texture analyzer (TA. XT Plus, Stable Micro Systems, Godalming, UK) equipped with a Warner–Bratzler shear (WBS) probe (HDP/BSW). The results were expressed in Newtons (N) as the mean of three consecutive measurements.

### 2.7. Analysis of Muscle Fatty Acid

Pectoral and leg muscle samples were collected at the 12th week of the experimental period, snap-frozen in liquid nitrogen, and stored at −80 °C for no longer than three months until fatty acid analysis. All extraction solvents contained 0.01% butylated hydroxytoluene (BHT) to prevent lipid oxidation during processing.

Fatty acids were analyzed by gas chromatography (GC) after conversion to fatty acid methyl esters (FAMEs) via lipid transesterification [20]. Approximately 0.2000 g of freeze-dried homogeneous muscle powder was transferred into a reaction tube. For derivatization, 3 mL formyl chloride, 2 mL of an internal standard solution (C11:0 in n-hexane, 0.1 mg/mL), and 1 mL n-hexane were added sequentially. The mixture was vortexed thoroughly, incubated in a water bath at 80 °C for 2 h, and subsequently cooled to room temperature. Then, 5 mL of 6% potassium carbonate solution was added and mixed well. The samples were centrifuged at 5000 rpm for 10 min at 4 °C, and the supernatant was collected for gas chromatographic determination.

Fatty acid composition was determined using an Agilent 7890B gas chromatograph equipped with a flame ionization detector (FID) (Agilent Technologies, Santa Clara, CA, USA). Separation was achieved using an HP-88 capillary column (30 m × 0.25 mm i.d., 0.25 µm film thickness). Nitrogen was used as the carrier gas at a flow rate of 1 mL/min, with a split ratio of 30:1 and an injection volume of 1 µL. Fatty acid content and composition were quantified using the internal standard method. The results were expressed as grams per kilogram (g/kg) of muscle tissue.

### 2.8. Calculations and Statistical Analysis

Data were analyzed by one-way ANOVA using GLM procedures of SAS v. 9.4 (SAS Institute Inc., Cary, NC, USA). The model included the treatment effect, and the pen presented the experimental unit for growth performance, while individual chickens were the experimental units for the other parameters. Treatment comparisons were performed using Duncan’s multiple range test for multiple tests. All values are presented as means ± standard errors of the mean (SEM). Differences were considered statistically significant at *p* < 0.05.

## 3. Results

### 3.1. Effects of Dietary Treatments Growth Performance

As shown in Table 3, dietary flaxseed supplementation had no significant effect on the FBW and ADG of Beijing-you chickens (*p* > 0.05), although numerically higher values were observed in the supplemented groups compared with the control.

### 3.2. Effects of Dietary Treatments Plasma Biochemical Parameters

Table 4 shows that, compared with the control group, dietary flaxseed supplementation significantly reduced plasma AST and ALT concentrations (*p* < 0.05). The lowest AST and ALT values were observed in the 10% and 6% flaxseed groups, respectively. Flaxseed supplementation also exerted a significant regulatory effect on plasma lipid metabolism (*p* < 0.05). Compared with the control group, birds fed flaxseed diets exhibited significantly lower total cholesterol (TC) and triglyceride (TG) levels (*p* < 0.05), accompanied by a significant increase in high-density lipoprotein cholesterol (HDL-C) concentration (*p* < 0.05). Notably, when the dietary flaxseed level exceeded 4%, TC and TG levels showed a significant decline relative to the control group (*p* < 0.05). However, no significant differences in TC or TG concentrations were detected among the high-dose flaxseed groups (6%, 8%, and 10%) (*p* > 0.05). Flaxseed supplementation had no significant effect on LDL-C or non-esterified fatty acids (NEFA), although numerical differences were observed. The lowest LDL-C concentration occurred at 6% flaxseed inclusion, whereas the highest NEFA concentration was observed at 2% flaxseed inclusion.

### 3.3. Effects of Dietary Treatments Meat Quality

Table 5 presents the effects of flaxseed supplementation on pectoral muscle quality in Beijing-you chickens. Compared with the control group, dietary inclusion of 2% flaxseed significantly reduced drip loss (*p* < 0.05). Flaxseed supplementation had no significant effects on muscle pH or shear force (*p* > 0.05), with differences observed only at the numerical level. Numerically, the highest pH value and the lowest shear force were recorded in birds fed the 6% flaxseed diet.

### 3.4. Effects of Dietary Treatments Muscle Fatty Acid

Graded flaxseed levels did affect the fatty acid composition in pectoral and leg muscles of Beijing-you chicken (Table 6). In the pectoral muscles, increasing the addition level of flaxseed led to a significant increase in the content of ω-3 PUFAs, while the content of C18:2n-6 and C20:4n-6 among ω-6 PUFAs were significantly decreased (*p* < 0.05). Furthermore, it was found that when the addition level of flaxseed exceeded 4%, no significant differences in fatty acid composition were detected among the different treatment groups (*p* > 0.05). The content of ω-3 PUFAs in leg muscle was significantly higher at 5.70 g/kg when fed 8% flaxseed (*p* < 0.05). With the increase in the dietary addition level of flaxseed, the content of EPA in the pectoral muscles and leg muscles significantly increased (*p* < 0.05), while the ω-6/ω-3 PUFAs in both muscles significantly decreased (*p* < 0.05). The order of deposition of ω-3 PUFAs in pectoral and leg muscles was ALA > DHA > EPA, and the difference in DHA, total SFA (saturated fatty acids), total MUFA (monounsaturated fatty acids) and total fatty acids content between groups was not significant (*p* > 0.05).

### 3.5. Effects of Dietary Treatments Omega-3 Fatty Acids Accumulation

Table 7 and Table 8 show the effects of different flaxseed feeding durations on fatty acid deposition in Beijing-you chickens. The accumulation of fatty acids in both pectoral and leg muscles increased significantly with duration, with feeding duration exerting a highly significant effect (*p* < 0.001). This time-dependent enrichment was most pronounced in the high-flaxseed groups (T4 and T5), which exhibited significantly higher total fatty acid levels compared to all other groups (*p* < 0.001).

## 4. Discussion

### 4.1. Discussion of Growth Performance

PUFAs play a crucial role in maintaining inflammatory balance, brain function, and cholesterol metabolism, thereby contributing to the prevention of various diseases such as cancer, osteoarthritis, and autoimmune disorders [21]. However, their health benefits are largely determined by the dietary ω-6 to ω-3 PUFA ratio, with a lower ratio (e.g., ≤4:1) being widely recommended for optimal health [22]. An excessively high ω-6/ω-3 ratio, which is common in modern diets, disturbs this balance and has been associated with an increased risk of various diseases, thereby underscoring the growing need for ω-3 enrichment. Functional poultry products can serve as a safe and reliable dietary source of ω-3 PUFAs for human consumption. Incorporating flaxseed into poultry diets has been demonstrated to be an effective strategy for producing ω-3 PUFAs-enriched functional products, in line with current nutritional recommendations [23,24,25]. Nevertheless, the presence of antinutritional factors limits the inclusion level of flaxseed in livestock and poultry diets [26,27]. Previous studies observed no significant differences in growth performance when 10% flaxseed was incorporated into broiler diets [28,29,30]. Similarly, no significant differences were found in the present study with flaxseed supplementation in growth performance. Although T2 and T3 groups showed numerical higher FBW and ADG than the control group, the lack of significance may be partly attributable to the relatively small sample size per treatment group (n = 8) in the present trial, which may have limited the statistical power to detect subtle differences. Therefore, further confirmation using larger sample sizes is warranted in future studies. Other studies have indicated that diets rich in ALA can promote growth and improve feed conversion ratio in laying hens [31] and pigs [32], while high-ALA diets have also been shown to enhance growth rate and increase the deposition of long-chain ω-3 PUFAs in the pectoral muscle of broiler chickens [33]. Therefore, dietary flaxseed supplementation suggests the potential to enhance growth performance and nutrient utilization efficiency in poultry.

### 4.2. Discussion of Plasma Biochemical Parameters

Plasma AST and ALT levels are commonly used biomarkers for evaluating liver function [34], with elevated levels indicating potential hepatic injury [35]. In the present study, compared with the control group, flaxseed supplementation (especially in T3 and T5) significantly reduced ALT and AST levels, suggesting a potential hepatoprotective effect. This finding aligns with the results of Manimurugan et al. [36], who reported that ω-3 PUFAs abundant in flaxseed can alleviate hepatic damage by inhibiting the NF-κB signaling pathway and suppressing the release of proinflammatory cytokines such as TNF-α, IL-1β, and IL-6 [37]. Moreover, dietary flaxseed supplementation markedly reduced plasma TC and TG in T3–T5, while HDL-C was significantly increased in T3–T5. In addition, NEFA concentrations were elevated in T1–T4 but decreased in T5. These results indicate that flaxseed exerts a lipid-modulating effect, putatively attributed to its high ω-3 PUFA content. ω-3 PUFAs can enhance low-density lipoprotein receptor (LDLR) activity, stimulate lipoprotein lipase (LPL) activity, and reduce apolipoprotein C-III levels, thereby facilitating lipid clearance from circulation [38]. Additionally, ALA from flaxseed acts as a signaling molecule that regulates the expression of genes associated with fatty acid metabolism through the activation of PPARα [39]. Lignans present in flaxseed have also been reported to reduce plasma TC levels and mitigate the progression of atherosclerosis, while the abundant dietary fiber may interfere with bile acid reabsorption and promote hepatic cholesterol utilization for bile acid synthesis, leading to a further decline in plasma cholesterol concentrations [40,41]. These results are consistent with the findings of Attia et al. [42]. Collectively, these data suggest that flaxseed supplementation at levels above 4% is not detrimental to avian hepatic health and can improve serum lipid profiles through multiple metabolic pathways.

### 4.3. Discussion of Meat Quality

Muscle pH, shear force, and drip loss are key indicators for evaluating the physicochemical properties of meat quality. The pH value affects the shelf life and storage stability of chicken meat, while drip loss reflects the water retention capacity of muscle. Lower drip loss indicates stronger water retention and better juiciness. Shear force serves as a measure of meat tenderness, with lower values corresponding to higher tenderness. Chickens receiving flaxseed supplementation exhibited significantly lower drip loss than the control group, whereas muscle pH and shear force were only numerically reduced, indicating a potential improvement in water retention capacity. This improvement may be attributed to the high PUFAs content of flaxseed, which alters the fluidity and permeability of muscle cell membranes and enhances membrane oxidative stability, thereby influencing water retention and tenderness [43]. Furthermore, antioxidant peptides derived from flaxseed can effectively scavenge free radicals and inhibit oxidative cross-linking between myosin and actin, maintaining the structural integrity and functional activity of proteins, thus further enhancing meat tenderness [44]. It has also been demonstrated that the water retention capacity of meat products is closely associated with the spatial conformation of myofibrillar proteins and electrostatic interactions between myofilaments [45]. Anjum et al. reported that in broiler chickens, a 15% flaxseed inclusion level could reduce the sensory acceptability of meat, due to significantly increasing ω-3 PUFAs content [46]. Similarly, Ali et al. observed that in broiler chicks, dietary inclusion of 4% flaxseed oil had no effect on meat pH or shear force but improved tenderness and ω-3 PUFAs levels [47]. Consistent with these findings, our results indicate that flaxseed inclusion levels exceeding 2% can enhance juiciness without compromising fundamental meat quality characteristics.

### 4.4. Discussion of Muscle Fatty Acid

This study demonstrated that increasing dietary flaxseed supplementation up to 8% significantly elevated the concentrations of ALA and EPA in the muscle tissues of Beijing-you chickens, whereas DHA levels remained unchanged after the 12-week study. The absence of a DHA increase may be attributed to the limited conversion efficiency of ALA to DHA, which is primarily constrained by low Δ-6 desaturase activity and competition with linoleic acid for the same enzymatic pathway [48]. In addition, although intramuscular fat was not determined in this study, it is possible that relatively high fat deposition could exert feedback inhibition on DHA synthesis [49]. With respect to deposition patterns, the accumulation of ω-3 PUFAs in both pectoral and leg muscles followed the order ALA > DHA > EPA, which is consistent with previous findings [50]. As dietary flaxseed inclusion increased, ALA content rose from 0.32 to 1.44 g/kg in pectoral muscle and from 1.23 to 5.12 g/kg in leg muscle. This dose-dependent increase directly demonstrates that dietary fatty acid composition influences muscle lipid profiles, consistent with findings in other monogastrics [40].

PUFAs accumulation was higher in leg than in pectoral muscle, which might be associated with potential differences in lipid content and lipid class composition. EPA and DHA tend to accumulate in tissues with higher fat content [51]. Consistent with this principle, leg muscle in poultry is generally reported to have a higher lipid content than pectoral muscle [52]. Moreover, ω-3 PUFAs in poultry are primarily incorporated into phospholipids [53], whereas ALA is preferentially esterified into triacylglycerols, a process more active in leg muscle owing to its higher lipid content and metabolic profile [54]. In addition, the limited conversion of ALA to DHA due to low Δ-6 desaturase activity further favors direct deposition rather than metabolic conversion [55]. Consequently, the observed pattern of ALA deposition and constrained DHA accumulation in leg muscle may be associated with a potentially higher triacylglycerol content.

### 4.5. Discussion of Omega-3 Fatty Acids Accumulation

The accumulation of omega-3 fatty acids described in this section was assessed in a parallel, independently sampled cohort of birds to avoid confounding the time-course analysis with terminal dose–response comparisons. The duration of dietary flaxseed supplementation significantly affected ω-3 PUFA deposition in Beijing-you chickens, with leg muscle showing a more pronounced time-dependent response than pectoral muscle. Prolonged feeding increased the gradual accumulation of ω-3 PUFAs, particularly EPA, whereas DHA showed limited incremental deposition, consistent with the restricted metabolic conversion capacity in poultry [56]. Accordingly, ω-3 PUFA levels in leg muscle increased progressively with feeding duration, confirming its greater storage potential compared with pectoral muscle.

In contrast, pectoral muscle is composed predominantly of fast-twitch fibers, which are characterized by lower mitochondrial density, limited β-oxidation capacity, and a primary reliance on glycolytic energy metabolism [55]. Such a constrained fatty acid–oxidative environment not only restricts the efficient metabolism of ALA but may also favor its esterification into storage triglycerides rather than its further desaturation and elongation to EPA and DHA. These metabolic characteristics likely explain the observed relationship between flaxseed level and feeding duration in breast muscle, where EPA levels reached their maximum only after 12 weeks at an 8% inclusion level. Collectively, from a practical perspective, optimizing both inclusion level (e.g., 8%) and feeding duration (e.g., 12 weeks) appears essential for maximizing ω-3 PUFAs enrichment, which is predominantly attributed to the accumulation of ALA.

### 4.6. Study Limitations and Practical Considerations

While this study clarifies distinct patterns of ω-3 PUFAs deposition in poultry meat, the limitations of flaxseed-based enrichment must be acknowledged. Firstly, the oxidative stability of the enriched muscle was not assessed. The elevated levels of ALA and EPA increase susceptibility to lipid oxidation, which can lead to rancidity, discoloration, and nutrient loss during storage, potentially compromising meat quality and shelf life. Secondly, the omission of sex as an analytical variable represents a key methodological limitation, and all treatment effects reported herein are interpreted with this constraint in mind. Future studies adopting sex-separated experimental designs are recommended to elucidate the potential modulating effects of sex on ω-3 PUFAs deposition. Thirdly, the statistical power of this study was constrained by the moderate sample size per treatment group. Consequently, Duncan’s multiple range test was employed for post hoc comparisons, and its limitations in controlling Type I error are acknowledged. Results with marginal significance are interpreted with particular caution. Fourthly, the effects observed at the end of the 12-week feeding period require careful interpretation. Fatty acid profiles were evaluated using two independently sampled groups of birds. Although flaxseed supplementation generally increased the contents of DHA and total ω-3 PUFAs in pectoral muscle and EPA in leg muscle, the magnitude and statistical significance of these effects were not fully consistent between sampling groups. Such variability is likely attributable to individual differences in fatty acid metabolism and the distinct lipid deposition patterns of different muscle types, underscoring that the effects are modest and muscle-specific. Finally, the practical application of high flaxseed inclusion (8–10%) involves several constraints beyond nutritional efficacy. These include the need for cost-adding heat treatments to reduce antinutritional factors, potential impacts on feed palatability and growth performance at a commercial scale, and the risk that high ω-3 PUFAs levels may alter meat flavor or, through enhanced oxidative processes, induce off-flavors, thereby reducing consumer acceptance.

## 5. Conclusions

In conclusion, dietary flaxseed supplementation improved certain meat quality and plasma lipid traits and increased ω-3 PUFAs content in the muscle of Beijing-you chickens without impairing growth performance. The most pronounced ω-3 PUFAs enrichment was generally achieved at an 8% inclusion level over 12 weeks. However, the response was moderate and varied between muscle types and sampling groups. Overall, flaxseed shows potential for enhancing ω-3 PUFAs levels in this breed, albeit with variable efficacy.

## Figures and Tables

**Table 1:** Nutritional composition of flaxseed (as-fed basis).

Components	
Moisture (%)	6.77
Ash (%)	2.95
Crude Fibre (%)	5.58
Crude Protein (%)	21.16
Ether Extract (%)	38.10
α-linolenic acid (C18:3n-3; ALA) (g/kg)	22.72
Eicosapentaenoic acid (C20:5n-3; EPA) (g/kg)	ND ^1^
Docosahexaenoic acid (C22:6n-3; DHA) (g/kg)	ND
Omega-3 polyunsaturated fatty acids ^2^ (ω-3 PUFAs) (g/kg)	22.72
Total Fatty Acids ^3^ (g/kg)	293.72

^1^ ND = Not Detected, detection value below detection level. ^2^ ω-3 PUFAs, including α-linolenic acid, Eicosapentaenoic acid, and Docosahexaenoic acid. ^3^ Total Fatty Acids represent the sum of all identified fatty acids in the sample.

**Table 2:** Ingredient and nutritional composition of the diets (as-fed basis).

Components	CT	T1	T2	T3	T4	T5
Corn (%)	62.98	61.75	60.7	59.46	58.19	56.81
Soybean meal (%)	31.75	31.35	30.85	30.40	30.00	29.60
Flaxseed (%)	0	2.00	4.00	6.00	8.00	10.00
Soybean oil (%)	1.82	1.50	1.00	0.80	0.60	0.40
Limestone (%)	1.36	1.31	1.26	1.20	1.12	1.10
Dicalcium phosphate (%)	1.30	1.30	1.30	1.30	1.30	1.30
Salt (%)	0.35	0.35	0.35	0.35	0.35	0.35
L-Lysine HCL (%)	0.05	0.05	0.05	0.05	0.05	0.05
DL-methionine (%)	0.07	0.07	0.07	0.07	0.07	0.07
Trace elements (%)	0.20	0.20	0.20	0.20	0.20	0.20
Vitamins (%)	0.02	0.02	0.02	0.02	0.02	0.02
Choline chloride (%)	0.10	0.10	0.10	0.10	0.10	0.10
Nutrition composition (%)						
Metabolizable Energy (ME; MJ/Kg)	12.04	12.04	11.96	11.96	11.96	11.96
Crude protein (%)	18.87	19.36	19.52	20.09	19.11	20.29
Ether extract (%)	3.50	3.74	4.67	5.61	5.74	5.82
Calcium (%)	0.87	0.87	0.87	0.88	0.87	0.88
Available phosphorus (%)	0.35	0.35	0.35	0.36	0.36	0.36
Total Lysine (%)	1.00	0.99	0.99	0.98	0.98	0.98
Total Methionine (%)	0.34	0.34	0.34	0.34	0.34	0.34
ALA (g/kg)	1.52	4.30	8.95	13.87	15.16	21.21
EPA (g/kg)	ND ^1^	ND	ND	ND	ND	ND
DHA (g/kg)	ND	ND	ND	ND	ND	ND
ω-3 PUFAs ^2^ (g/kg)	1.52	4.30	8.95	13.87	15.16	21.21
ω-6 PUFAs ^3^ (g/kg)	22.20	18.57	23.10	23.23	21.42	22.18
ω-6/ω-3 PUFAs	14.64	4.32	2.58	1.68	1.41	1.05
Total fatty acids ^4^ (g/kg)	44.84	43.36	57.11	63.58	62.76	69.31

^1^ ND = Not Detected, detection value below detection level. ^2^ ω-3 PUFAs, including α-linolenic acid, Eicosapentaenoic acid, and Docosahexaenoic acid. ^3^ ω-6 polyunsaturated fatty acids (ω-6 PUFAs), including Linoleic acid (C18:2n-6), γ-linolenic acid (C18:3n-6) and Dihomo-γ-linolenic acid (C20:3n-6). ^4^ Total Fatty Acids represent the sum of all identified fatty acids in the sample.

**Table 3:** Effects of flaxseed supplementation on final body weight of Beijing-you chickens.

	CT	T1	T2	T3	T4	T5	SEM	*p*-Value
FBW (g)	1770	1782	1833	1882	1795	1819	51	0.720
ADG (g)	13.71	14.17	14.61	15.17	14.15	14.48	0.56	0.594

FBW—final body weight; ADG—average daily gain; CT, control group; T1, 2% flaxseed supplementation; T2, 4% flaxseed supplementation; T3, 6% flaxseed supplementation; T4, 8% flaxseed supplementation; T5, 10% flaxseed supplementation. The absence of superscript letters within a row indicates no significant difference among treatments.

**Table 4:** Effects of flaxseed supplementation on plasma parameters of Beijing-you chickens.

Item	CT	T1	T2	T3	T4	T5	SEM	*p*-Value
AST (IU/L)	32.83 ^a^	33.21 ^a^	27.64 ^ab^	27.90 ^ab^	31.87 ^a^	24.21 ^b^	1.78	<0.001
ALT (IU/L)	8.89 ^a^	6.79 ^ab^	7.02 ^ab^	6.04 ^b^	6.85 ^ab^	7.39 ^ab^	0.61	0.005
TC (mmol/L)	9.08 ^a^	9.23 ^a^	6.92 ^ab^	7.98 ^bc^	8.83 ^c^	8.24 ^c^	0.79	<0.001
TG (mmol/L)	0.35 ^a^	0.11 ^a^	0.32 ^ab^	0.25 ^bc^	0.21 ^c^	0.22 ^c^	0.05	<0.001
HDL-C (mmol/L)	2.24 ^bc^	2.57 ^abc^	2.15 ^c^	2.90 ^a^	3.01 ^a^	2.82 ^ab^	0.17	0.001
LDL-C (mmol/L)	0.57	0.50	0.61	0.44	0.56	0.57	0.10	0.668
NEFA (mmol/L)	0.53	0.63	0.55	0.58	0.56	0.49	0.07	0.702

AST—aspartate aminotransferase; ALT—alanine aminotransferase; TC—total cholesterol; TG—triglyceride; HDL-C—high-density lipoprotein cholesterol; LDL-C—low-density lipoprotein cholesterol; NEFA—non-esterified fatty acids; CT, control group; T1, 2% flaxseed supplementation; T2, 4% flaxseed supplementation; T3, 6% flaxseed supplementation; T4, 8% flaxseed supplementation; T5, 10% flaxseed supplementation. ^a, b, c^ Values within the same row bearing different superscripts are considered significantly different (*p* < 0.05).

**Table 5:** Effects of flaxseed supplementation on pectoral muscle meat quality of Beijing-you chickens.

Item	CT	T1	T2	T3	T4	T5	SEM	*p*-Value
pH	5.80	5.80	5.71	5.83	5.79	5.78	0.819	0.106
Drip loss (%)	4.47 ^a^	2.94 ^b^	3.60 ^ab^	4.01 ^ab^	3.72 ^ab^	3.24 ^ab^	0.744	0.022
Shear force (N)	18.66	16.84	17.25	16.19	18.52	17.82	3.312	0.413

CT, control group; T1, 2% flaxseed supplementation; T2, 4% flaxseed supplementation; T3, 6% flaxseed supplementation; T4, 8% flaxseed supplementation; T5, 10% flaxseed supplementation. ^a, b^ Values within the same row bearing different superscripts are considered significantly different (*p* < 0.05).

**Table 6:** Effect of flaxseed supplementation on fatty acids composition in muscle of Beijing-you chickens.

Item, g/kg	CT	T1	T2	T3	T4	T5	SEM	*p*-Value
pectoral muscle								
C14:0	0.06	0.07	0.04	0.04	0.03	0.03	0.01	0.395
C16:0	3.17	3.64	2.47	2.24	1.83	2.09	0.56	0.327
C16:1n-7	0.23	0.31	0.19	0.12	0.10	0.10	0.07	0.494
C17:0	0.05	0.05	0.04	0.03	0.03	0.04	0.01	0.365
C18:0	1.48	1.74	1.24	1.16	1.04	1.18	0.20	0.266
C18:1n-9	4.15	4.89	3.32	2.82	2.24	2.96	0.89	0.484
C18:2n-6	3.98 ^ab^	4.38 ^a^	2.98 ^ab^	2.82 ^ab^	2.16 ^b^	2.90 ^ab^	0.66	0.046
C18:3n-6	0.04	0.03	0.04	0.03	0.02	0.02	0.01	0.399
ALA	0.32	0.70	0.74	0.89	0.83	1.44	0.21	0.052
C20:1n-9	0.03	0.04	0.02	0.02	0.01	0.02	0.01	0.273
C20:2n-6	0.05	0.05	0.03	0.04	0.03	0.03	0.01	0.139
C20:3n-6	0.10	0.10	0.10	0.11	0.09	0.09	0.01	0.766
C20:4n-6	0.84 ^a^	0.86 ^a^	0.73 ^ab^	0.64 ^b^	0.60 ^b^	0.57 ^b^	0.04	<0.001
C24:1n-9	0.16	0.10	0.08	0.07	0.05	0.04	0.01	0.075
EPA	0.02 ^b^	0.05 ^b^	0.07 ^a^	0.09 ^a^	0.09 ^a^	0.10 ^a^	0.01	<0.001
DHA	0.28	0.36	0.35	0.32	0.28	0.37	0.03	0.246
ω-3 PUFAs	0.61 ^b^	1.11 ^ab^	1.16 ^ab^	1.30 ^ab^	1.20 ^ab^	1.91 ^a^	0.23	0.039
ω-6 PUFAs	4.96	5.37	3.86	3.59	2.86	3.58	0.76	0.342
ω-6/ω-3 PUFAs	7.62 ^a^	4.73 ^b^	3.46 ^bc^	2.76 ^cd^	2.55 ^cd^	1.98 ^d^	0.28	<0.001
Total SFA ^1^	3.58	3.76	3.03	2.80	2.90	2.61	0.522	0.581
Total MUFA ^2^	7.25	7.98	6.74	6.24	6.51	6.42	1.112	0.906
Total Fatty Acids ^3^	10.83	11.75	9.77	9.04	9.41	9.03	1.571	0.831
leg muscle								
C14:0	0.26	0.23	0.18	0.23	0.19	0.11	0.05	0.441
C16:0	9.61	9.00	7.51	8.39	7.18	4.49	1.58	0.411
C16:1n-7	1.34	1.05	0.96	0.88	0.99	0.27	0.27	0.252
C17:0	0.14	0.15	0.12	0.15	0.12	0.11	0.02	0.794
C18:0	3.74	3.90	3.32	3.72	3.13	2.76	0.50	0.660
C18:1n-9	13.54	12.85	11.53	12.40	10.70	7.16	2.42	0.599
C18:2n-6	14.42	13.43	11.38	13.61	10.90	8.81	2.31	0.642
C18:3n-6	0.24	0.19	0.17	0.19	0.17	0.13	0.03	0.453
ALA	1.23 ^b^	2.22 ^ab^	3.02 ^ab^	4.89 ^a^	5.12 ^a^	4.77 ^a^	0.67	0.003
C20:1n-9	0.11	0.10	0.09	0.10	0.07	0.05	0.02	0.622
C20:2n-6	0.14	0.13	0.10	0.11	0.09	0.08	0.02	0.137
C20:3n-6	0.13	0.10	0.10	0.13	0.11	0.09	0.02	0.462
C20:4n-6	1.29 ^a^	1.14 ^ab^	1.04 ^bc^	0.98 ^bc^	0.93 ^c^	0.88 ^c^	0.04	<0.001
C24:1n-9	0.31 ^a^	0.22 ^b^	0.18 ^bc^	0.16 ^bc^	0.15 ^bc^	0.12 ^c^	0.02	<0.001
EPA	0.04 ^c^	0.07 ^c^	0.11 ^bc^	0.15 ^ab^	0.19 ^a^	0.17 ^ab^	0.02	<0.001
DHA	0.34	0.39	0.39	0.39	0.39	0.42	0.03	0.659
ω-3 PUFAs	1.61 ^f^	2.67 ^e^	3.50 ^d^	5.43 ^b^	5.70 ^a^	5.36 ^c^	0.69	0.002
ω-6 PUFAs	16.08	14.86	12.69	14.89	12.10	9.90	2.37	0.591
ω-6/ω-3 PUFAs	9.97 ^a^	5.53 ^b^	3.65 ^c^	2.63 ^cd^	2.23 ^d^	1.86 ^d^	0.19	<0.001
Total SFA ^1^	10.53	10.71	9.70	8.22	7.96	6.66	1.41	0.405
Total MUFA ^2^	26.31	25.61	26.17	21.94	22.49	20.20	4.07	0.869
Total Fatty Acids ^3^	36.84	36.32	35.86	30.15	30.45	26.85	5.47	0.768

CT, control group; T1, 2% flaxseed supplementation; T2, 4% flaxseed supplementation; T3, 6% flaxseed supplementation; T4, 8% flaxseed supplementation; T5, 10% flaxseed supplementation. ^1^ Total SFA represent the sum of all saturated fatty acids. ^2^ Total MUFA represent the sum of all monounsaturated fatty acids. ^3^ Total Fatty Acids represent the sum of all identified fatty acids in the sample. ^a, b, c, d, e, f^ Values within the same row bearing different superscripts are considered significantly different (*p* < 0.05).

**Table 7:** Effect of different feeding durations of flaxseed on fatty acids accumulation in pectoral muscle of Beijing-you chickens.

Item, g/kg	CT	T1	T2	T3	T4	T5	SEM	*p*-Value
EPA								
2w	0.020 ^c^	0.025 ^c^	0.038 ^b^	0.055 ^a^	0.063 ^a^	0.062 ^a^	0.004	<0.001
4w	0.015 ^d^	0.027 ^d^	0.050 ^c^	0.065 ^b^	0.073 ^ab^	0.080 ^a^	0.004	<0.001
6w	0.017 ^d^	0.030 ^cd^	0.045 ^bc^	0.053 ^ab^	0.063 ^a^	0.067 ^a^	0.004	<0.001
8w	0.013 ^c^	0.032 ^c^	0.053 ^b^	0.073 ^ab^	0.073 ^ab^	0.092 ^a^	0.006	<0.001
10w	0.018 ^c^	0.030 ^c^	0.064 ^b^	0.073 ^b^	0.092 ^ab^	0.118 ^a^	0.009	<0.001
12w	0.020 ^d^	0.045 ^cd^	0.072 ^bc^	0.087 ^b^	0.124 ^a^	0.098 ^ab^	0.009	<0.001
DHA								
2w	0.097 ^d^	0.143 ^c^	0.157 ^bc^	0.152 ^bc^	0.187 ^ab^	0.195 ^a^	0.011	<0.001
4w	0.180 ^bc^	0.163 ^c^	0.210 ^ab^	0.242 ^a^	0.228 ^a^	0.240 ^a^	0.014	<0.001
6w	0.190 ^c^	0.200 ^bc^	0.248 ^ab^	0.242 ^abc^	0.275 ^a^	0.225 ^abc^	0.016	0.015
8w	0.180 ^c^	0.235 ^b^	0.248 ^ab^	0.253 ^ab^	0.295 ^a^	0.252 ^ab^	0.016	0.011
10w	0.214 ^c^	0.275 ^bc^	0.384 ^a^	0.338 ^ab^	0.378 ^a^	0.377 ^a^	0.022	0.001
12w	0.262 ^ab^	0.360 ^a^	0.347 ^a^	0.318 ^ab^	0.219 ^b^	0.368 ^a^	0.033	0.022
ω-3PUFAs								
2w	0.210 ^c^	0.423 ^bc^	0.487 ^bc^	0.707 ^ab^	0.990 ^a^	0.902 ^a^	0.097	<0.001
4w	0.245 ^d^	0.417 ^dc^	0.908 ^bdc^	1.430 ^ab^	1.100 ^abc^	1.778 ^a^	0.148	0.001
6w	0.370	0.674	0.922	0.947	1.315	1.420	0.182	0.079
8w	0.410	0.533	1.383	1.875	1.673	1.635	0.269	0.050
10w	0.707	0.887	1.714	1.208	1.380	2.853	0.386	0.089
12w	0.732	1.107	1.157	1.298	3.394	1.910	0.420	0.057

CT, control group; T1, 2% flaxseed supplementation; T2, 4% flaxseed supplementation; T3, 6% flaxseed supplementation; T4, 8% flaxseed supplementation; T5, 10% flaxseed supplementation. ^a, b, c, d^ Values within the same row bearing different superscripts are considered significantly different (*p* < 0.05).

**Table 8:** Effect of different feeding durations of flaxseed on fatty acids accumulation in leg muscle of Beijing-you chickens.

Item, g/kg	CT	T1	T2	T3	T4	T5	SEM	*p*-Value
EPA								
2w	0.043 ^d^	0.047 ^d^	0.095 ^c^	0.117 ^bc^	0.143 ^ab^	0.160 ^a^	0.009	<0.001
4w	0.023 ^d^	0.060 ^cd^	0.105 ^bc^	0.138 ^ab^	0.153 ^ab^	0.172 ^a^	0.013	<0.001
6w	0.037 ^c^	0.082 ^bc^	0.133 ^ab^	0.118 ^ab^	0.153 ^a^	0.165 ^a^	0.017	0.003
8w	0.030 ^d^	0.082 ^cd^	0.138 ^bc^	0.156 ^abc^	0.193 ^ab^	0.228 ^a^	0.017	<0.001
10w	0.043 ^d^	0.073 ^cd^	0.105 ^bcd^	0.112 ^bc^	0.158 ^ab^	0.187 ^a^	0.017	0.001
12w	0.037	0.070	0.130	0.153	0.193	0.362	0.047	0.104
DHA								
2w	0.207 ^d^	0.247 ^cd^	0.295 ^bc^	0.287 ^bc^	0.337 ^ab^	0.358 ^a^	0.016	<0.001
4w	0.263 ^b^	0.280 ^b^	0.373 ^a^	0.403 ^a^	0.385 ^a^	0.412 ^a^	0.024	0.001
6w	0.333 ^c^	0.347 ^bc^	0.437 ^ab^	0.382 ^abc^	0.463 ^a^	0.403 ^abc^	0.030	0.046
8w	0.293 ^b^	0.375 ^a^	0.428 ^a^	0.418 ^a^	0.458 ^a^	0.443 ^a^	0.024	0.011
10w	0.323 ^c^	0.427 ^abc^	0.527 ^a^	0.403 ^bc^	0.467 ^ab^	0.455 ^ab^	0.027	0.021
12w	0.342	0.387	0.407	0.385	0.387	0.456	0.034	0.358
ω-3PUFAs								
2w	1.207 ^c^	1.965 ^bc^	2.898 ^bc^	3.080 ^b^	4.873 ^a^	5.285 ^a^	0.387	<0.001
4w	0.977 ^b^	1.970 ^b^	3.713 ^a^	4.630 ^a^	4.083 ^a^	4.423 ^a^	0.440	<0.001
6w	1.720 ^c^	2.567 ^bc^	4.335 ^ab^	4.315 ^ab^	5.893 ^a^	5.927 ^a^	0.528	<0.001
8w	1.523 ^c^	2.993 ^bc^	4.852 ^ab^	6.008 ^a^	6.617 ^a^	7.055 ^a^	0.737	0.002
10w	1.657 ^d^	2.478 ^cd^	3.108 ^bcd^	3.980 ^bc^	4.398 ^b^	6.157 ^a^	0.444	<0.001
12w	1.610 ^b^	2.673 ^b^	4.547 ^ab^	5.425 ^ab^	5.703 ^ab^	8.360 ^a^	1.167	0.047

CT, control group; T1, 2% flaxseed supplementation; T2, 4% flaxseed supplementation; T3, 6% flaxseed supplementation; T4, 8% flaxseed supplementation; T5, 10% flaxseed supplementation. ^a, b, c, d^ Values within the same row bearing different superscripts are considered significantly different (*p* < 0.05).

## Data Availability

Data is contained within the article.

## Abbreviations

The following abbreviations are used in this manuscript:

ADFI	Average Daily Feed Intake
ADG	Average Daily Gain
ALA	α-linolenic Acid
ALT	Alanine Aminotransferase
AST	Aspartate Aminotransferase
CT	Control Group
DHA	Docosahexaenoic Acid
EPA	Eicosapentaenoic Acid
FBW	Final Body Weight
FCR	Feed Conversion Ratio
HDL-C	High-Density Lipoprotein Cholesterol
LAs	Linoleic Acids
LDL-C	Low-Density Lipoprotein Cholesterol
MUFAs	Monounsaturated Fatty Acids
NEFA	Non-Esterified Fatty Acids
PUFAs	Polyunsaturated Fatty Acids
TC	Total Cholesterol
TG	Triglyceride
ω-3 PUFAs	Omega-3 Polyunsaturated Fatty Acids
ω-6 PUFAs	Omega-6 Polyunsaturated Fatty Acids
SFA	saturated fatty acids
MUFA	monounsaturated fatty acids
